# Designing supply chains to meet the growing need of vaccines: evidence from four countries

**DOI:** 10.1186/s40545-021-00368-x

**Published:** 2021-09-29

**Authors:** Wendy Prosser, Cary Spisak, Benjamin Hatch, Joseph McCord, Marie Tien, Greg Roche

**Affiliations:** John Snow, Incorporated, 2733 Crystal Drive, 4th Floor, Arlington, VA 22202 USA

**Keywords:** Vaccine, Supply chain, System design

## Abstract

**Background:**

Immunization supply chains (iSCs) move vaccines from manufacturer to point of use with the added complexities of requiring cold chain and an increasing need for agility and efficiency to ensure vaccine quality and availability. Underperforming iSCs have been widely acknowledged as a key constraint to achieving high immunization coverage rates in low- and middle-income countries. This paper details the system design approach used to analyze the iSC network in Sierra Leone, Madagascar, Niger and Guinea and documents six lessons.

**Methodology:**

Between 2018 and 2020, these countries implemented the system design approach, involving four key steps: (1) advocate and introduce to engage stakeholders and prioritize identification of modeling scenarios; (2) collect data and plan analysis through document review and key informant interviews; (3) analyze system design scenarios using computer software modeling tools (LLamasoft’s Supply Chain Guru and AnyLogic's AnyLogistix) for optimization and simulation modeling as well as further analysis with Excel, Google maps, and OpenStreetMap; and (4) build consensus on optimized model and implementation roadmap using the Traffic Light Analysis tool and building on stakeholder input.

**Findings:**

Key lessons include the following: (1) define system design objectives based on country priorities; (2) establish consensus with stakeholders on scenarios to model; (3) modeling provides the evidence but not the answer; (4) costs should not be weighted above other decision criteria; (5) data collection—work smarter, not harder; (6) not all questions can be answered with a computer model.

**Discussion:**

A system design approach can identify changes to the design of the supply chain that can introduce efficiencies and improve reliability. This approach can be more effective when these lessons and principles are applied at the country level. The lessons from these four countries contribute to global thinking and best practices related to system design. The modeling and system design approach provides illustrative results to guide decision-makers. It does not give a "final answer", but compares and contrasts.

## Background

Public health supply chains move products from manufacturer to point of use through many distribution layers using various modes of transport and storage. Vaccines have the added complexity of requiring cold chain and more agility and efficiency to ensure product quality and availability. Underperforming immunization supply chains (iSC) have been widely acknowledged as a key constraint to achieving high immunization coverage rates in low- and middle-income countries [1, 2]. Many of these supply chains were designed more than 40 years ago, typically following administrative tiers, and now are considered outdated, inefficient, and unreliable. As a result, frequent stockouts hinder immunization coverage, excessive stock creates inefficiencies and waste, inaccurate forecasting can delay procurement and distribution, and non-functioning cold chain equipment (CCE) can diminish the quality of the vaccines.

As new vaccines are developed and immunization programs expand, there is ever-growing pressure to reduce wastage, improve efficiencies and reliability, and introduce agility to the iSC [3, 4]. Considerable investments and efforts have been made to reach that goal. Many entities, in both the public and private sectors, have used a system design approach to analyze options to improve supply chain performance.

This paper details the system design approach for the iSC network and lessons learned in Sierra Leone, Madagascar, Niger, and Guinea. Results of the analysis have been published elsewhere [5]. The authors were hired to undertake a system design evaluation in each country, with the goal of identifying iSC bottlenecks, assisting stakeholders in identifying system design changes that might help remove these bottlenecks, and modeling these changes with network optimization software to advise on the likely outcomes of each possible system design change. The objective of this paper is to document six lessons from these four countries and the system design approach used to ensure evidence-driven, forward-looking decisions to improve supply chain performance.

## Methodology

**Fig. 1.**
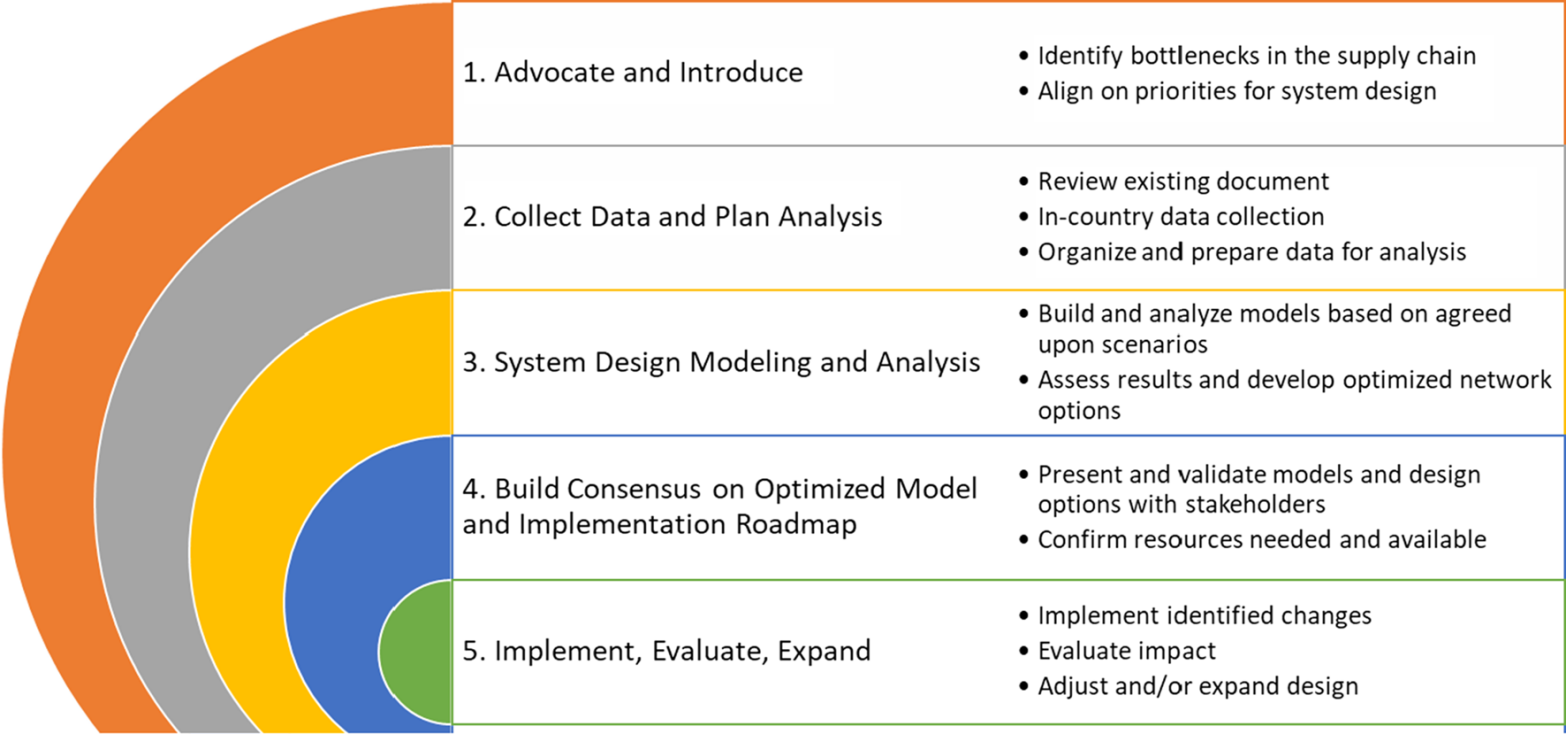
The steps in a system design approach

**Fig. 2:**
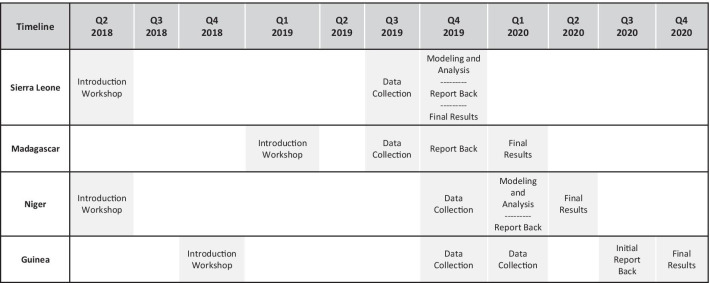
Timeline for system design activities in four countries

Between 2018 and 2020, these four countries implemented the system design approach (Fig. 1), which includes stakeholder engagement and the application of various modeling tools to quantitatively and qualitatively analyze the different components of the overall supply chain system and policies. The objective was to identify changes to component interaction to improve efficiency. The five-step approach allows stakeholders to evaluate different scenarios, pose “what if” questions, and measure the impact of proposed changes. The modeling results provide evidence that must be validated with stakeholders for a more informed decision on the iSC design [6–11] (Fig. 2). Each country undertook the following steps.

### Step 1: Advocate and introduce

A broad stakeholder workshop in each country using case studies to demonstrate possible benefits ensured understanding of the system design approach. Participants included representatives from each level of the health system, from national to sub-national to health facility (HF) level. As the focus was on the iSC, immunization managers and logisticians were the key champions throughout this process; however, as integration with other supply chains was also being explored, representatives from Central Medical Stores (CMS) and other health programs also participated in some of the meetings. Other partners and funders involved in the supply chain, such as UNICEF; WHO; nongovernmental organizations; Gavi, the Vaccine Alliance; and USAID (in the case of Madagascar), were also involved.

A key workshop activity was to clearly identify known iSC bottlenecks, gaps, and strengths to identify distinct modeling scenarios that could mitigate bottlenecks and would be modeled under Step 3. The process involved a review of the country’s immunization program, including program and supply chain performance and challenges. Through consensus building, participants agreed on a common definition of a highly performing supply chain as the ultimate goal of the system design approach. This approach provided a concrete foundation and a clear roadmap for next steps.

### Step 2: Collect data and plan analysis

Categories of data needed include cost; human resources; network structure; CCE type, capacity, and location; and details on vaccines and consumables in the immunization schedule (Table 1). In Sierra Leone and Niger, additional information was collected on the estimated volume of oxytocin distributed to the facilities qualified to dispense the product to explore the possibility of integrating oxytocin into the vaccine cold chain. Additionally, scenarios exploring the use of autonomous aerial vehicles (AAVs) in Madagascar and Guinea required a number of assumptions on the AAV type, number required, payload capacity, location of AAV hubs, whether a third party or the ministry of health would manage the AAVs, and which facilities would receive them.

**Table 1:** Data required for modeling

Cost	●Human resources: civil servant pay scale for health and warehouse staff ●Vehicle: fixed purchase, capacity, fuel, operating expenses, number of facilities covered per delivery ●Public transportation: average cost per trip ●Investment: building construction and CCE needed to set up a new location; additional vehicles for direct delivery, new routes; new technology (AAVs), training new/additional personnel ●Operating and fixed storage ●Maintenance and fuel for CCE ●Straight line depreciation for vehicles and CCE
Human resources	●Title, average number of staff, and time spent conducting logistics management tasks ●Title, average number of staff at warehouse ●Title, average number of staff who pickup/drop off commodities
Network structure and facility data	●Number and location of all HF types that provide vaccines (with GIS coordinates) ●Number and location of central, regional, district warehouses (with GIS coordinates) ●Current delivery frequency/schedule for each supply chain level
Cold chain equipment	●Number, model, capacity, functional status of CCE at each HF or warehouse
Vaccines and other commodities	●Vaccines for each location ●Volume for each location: oTarget population oImmunization schedule oTarget coverage ●New products for introduction or integration—all of the above needed

In each country, much of the data needed were available through existing sources such as the Cold Chain Equipment Inventory and Gap Analysis Tool, the EPI Logistics Forecasting Tool, and master facility lists. Additionally, a data collection tool was used in a sample of facilities and warehouses to collect information to build out and validate costing assumptions. In three countries, a stratified sample using the Central Limit Theorem guided the selection of facilities (urban/rural and harder to reach), visiting approximately two regions and 16–36 sites in each country. In Madagascar, a purposeful sample was used to ensure representation of the hardest-to-reach areas. Data collection through key informant interviews focused on estimating the average number of staff at each location, the hours spent on logistics tasks for vaccine management and picking up or delivering vaccines, the transportation normally used to collect or drop off commodities, and fixed and operating costs. Data collection also captured costs that are typically hidden from the central government, such as health workers paying out of pocket for public transportation to collect vaccines.

**Fig. 3:**
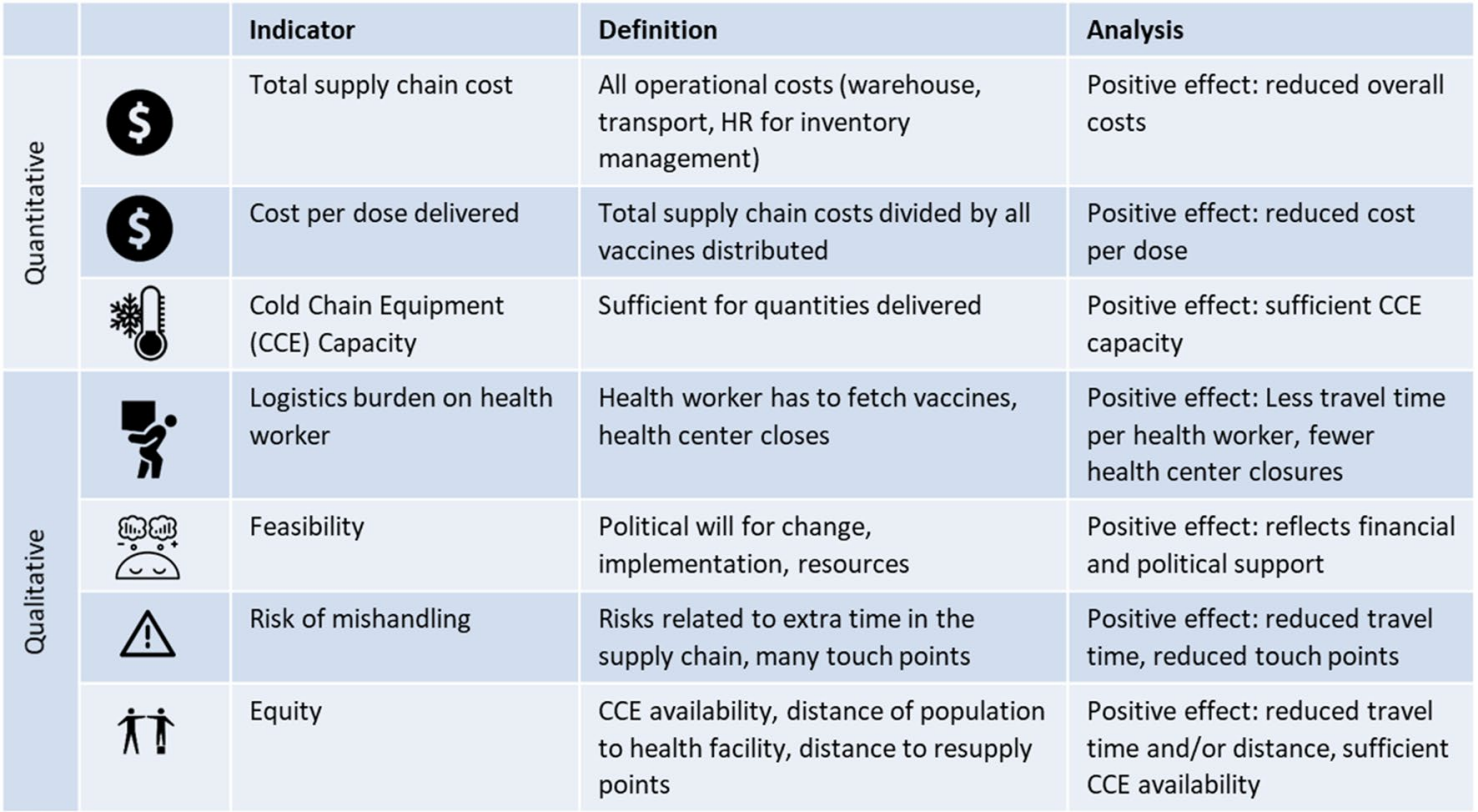
Decision criteria used for analysis

Stakeholders aligned on seven quantitative and qualitative key decision criteria that reflect the diverse aspects of supply chain performance and country priorities [12] (Fig. 3). Based on the results of the modeling, these criteria were analyzed for each scenario and compared to the baseline scenario.

### Step 3: Analyze system design scenarios using modeling tools

Computer software modeling tools, namely LLamasoft’s Supply Chain Guru and AnyLogic's AnyLogistix, were used for optimization and simulation modeling for the baseline of the current iSC and the scenarios chosen in each country. Optimization determines the lowest-cost constellation of current or theoretical resources, such as which facility should resupply from which warehouse, regardless of whether this constellation is possible with available resources. Simulation modeling, on the other hand, applies real-world conditions, such as constraints in the number of trucks and truck driver working hours that are not addressed by optimization [13]. The level of detail—such as facility and warehouse locations, CCE capacity, transportation, and warehousing costs per kilometer traveled or per cubic centimeter of product handled—necessary for the different supply chain scenarios is built into the model to evaluate the effects of policy or structural changes in terms of total system costs, costs per vaccine dose delivered, and volume throughput for each warehouse and HF. Additional analyses using Excel, Google maps, and OpenStreetMap were used to address aspects not directly included in the modeled scenarios, such as estimating the number of vehicles needed for different scenarios, cold chain capacity use, and change in the number of staff hours when shifting to direct delivery. For the purpose of this activity, all of these types of analysis fall under the broader term “modeling.”

### Step 4: Build consensus on optimized model and implementation roadmap

**Fig. 4:**
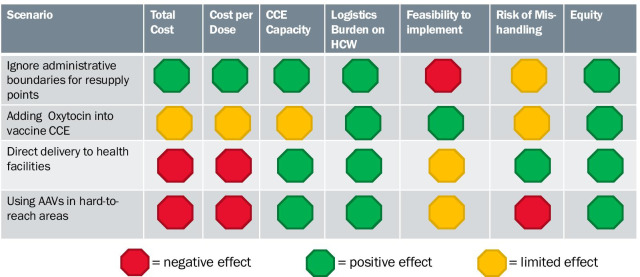
Sample of traffic light analysis tool

Results were presented to stakeholders using the Traffic Light Analysis Tool to assess the change in the indicators for each of the scenarios compared to the baseline scenario (Fig. 4). Green, yellow, and red symbols are used for each criteria to indicate a positive, limited, or negative effect, respectively. Each modeled scenario is evaluated in terms of quantitative changes (such as change in system cost or cost per dose delivered) and qualitative indicators (such as difficulty of implementation or equity considerations, defined as the change in the logistics burden placed on last-mile HFs). Qualitative indicators were evaluated under the traffic light scheme based on the appreciation of the project team (for example, changing distribution from quarterly to monthly for a system that requires last-mile HFs to travel to pick up their vaccines implies an increase in the logistics burden on those facilities and therefore a negative effect on equity overall; the equity indicator in this scenario would be labeled as red). At a final report-back workshop, stakeholders reviewed modeling results and agreed on priorities for implementation.

### Step 5: Implement, evaluate, and expand

This step involves implementing changes identified by the modeling and agreed upon by stakeholders. It was out of this activity’s scope.

## Findings

From this process emerged six key lessons that can be applied in other countries adopting a system design approach.

### Lesson 1: Define system design objectives based on country priorities

Based on the initial identification of bottlenecks and gaps in performance, stakeholders identified a few common priorities with the ultimate goal of improving immunization coverage rates that resonated across the four countries (Table 2). Stakeholders prioritized reducing operating costs and creating an efficient, equitable, and sustainable system. Any changes to the iSC should ensure immunization availability, particularly at last-mile HFs. Improving availability and use of CCE was another common theme. Finally, integration, either the “low-hanging fruit” of non-vaccine cold chain products such as oxytocin being included in the vaccine cold chain or the full-scale integration of vaccines into CMS was a common priority for three of the four countries.

**Table 2:** Country-identified bottlenecks in the current iSC and priorities for the system design approach

Bottlenecks	Priority
Sierra Leone	
●Inadequate workload balance ●Additional skills building needed ●Forecasts do not match consumption ●Insufficient funds for distribution	●Ensure availability of vaccines to all children ●Reduce operating costs/improve cost effectiveness ●Ensure CCE at all (or at least 80% of) HFs ●Optimize storage space ●Integrate with other cold chain products ●Consider different vaccine vial presentations ●Reach underserved/hard-to-reach areas (equity) ●Reduce time spent in logistics ●Guarantee distribution from district to HF ●Optimize district/regional stores and their linked network/transport routes ●Optimize transport system for district distribution and supervision
Niger	
●Insufficient transport, particularly at sub-national levels ●Security challenges in conflict-prone areas ●Human resource (HR) constraints in availability and skills	●Achieve a more reliable system ●Guarantee regular delivery of vaccines to the last mile ●Ensure quality of vaccines in sufficient quantity ●Optimize use of CCE ●Base supply chain decisions on actual data ●Integrate management system with qualified staff
Madagascar	
●Failure to use the regional administrative level for the iSC ●Unused regional cold rooms ●Antiquated facility-level CCE depend on propane tanks ●Inadequate funds and insufficient vehicles to support distribution ●Insufficient HR for iSC management ●Ad hoc district distribution to HFs	●Ensure an effective, equitable, and adaptable system ●Provide reliable data ●Reduce operating costs ●Increase immunization coverage ●Reduce expired vaccines ●Promote sustainability ●Upgrade CCE
Guinea	
●Lack of cold chain at regional level ●Insufficient cold chain capacity at districts and HFs ●Insufficient means of transport ●Insufficient HR capacity for CCE maintenance ●Inadequate budgeting and planning based on inaccurate population estimates	●Ensure an effective, equitable, and adaptable system ●Provide reliable data through computerized system ●Introduce cost savings ●Promote sustainability ●Decentralize to ensure all products are available at HF ●Integrate and provide high-quality services ●Ensure availability of CCE at all levels ●Allocate qualified and sufficient HR ●Establish regional cold chain depots

Sierra Leone stakeholders prioritized reducing the burden on health workers through direct delivery to HFs and were interested in considering different vial sizes to reduce missed opportunities for vaccination [14]. Stakeholders in Madagascar, Niger, and Guinea prioritized data availability for decision-making and were each closely exploring options for a fully integrated public health supply chain.

### Lesson 2: Establish consensus with stakeholders on scenarios to model

Stakeholders identified scenarios that reflected best practices in supply chain management while adapting to country context. Stakeholders in all four countries explored different delivery frequencies to sub-national stores and facilities (shifting to delivery to HFs every 2 months or quarterly instead of monthly) to optimize cold chain storage and transport. Stakeholders in Madagascar, Guinea, and Sierra Leone considered changing resupply points that ignore administrative boundaries to allow HFs to collect or receive vaccines from district resupply points to which they may not report administratively but that are geographically closer, thereby reducing travel time and costs. This reflects a private sector logistics company best practice.

Stakeholders in all four countries were interested in adjusting the number of supply chain layers, either by adding regional levels (to be more in-line with the administrative structure, as in Madagascar and Guinea) or by removing a layer to reduce the number of touch-points in the supply chain (Sierra Leone, Niger, and a variation of a scenario in Guinea). Removing a layer of the supply chain often reduces costs, decreases the time a product spends in the supply chain, and increases product availability [15]. In Guinea, Niger, and Sierra Leone there was also interest in direct delivery from the last resupply point at the district level to the HF to reduce health workers’ logistics burden of vaccine collection.

The final two common themes in the scenarios reflect the new AAV technology and the global push for supply chain integration. AAVs were included as a scenario in Madagascar and Guinea; interestingly, they were discussed in Niger, but ultimately not included as a scenario because AAVs would not be acceptable for use in the country’s high-risk security areas. Stakeholders in three countries considered some aspects of integration, with Sierra Leone and Niger most interested in including other cold chain products in the vaccine supply chain, and full integration of vaccines into the CMS considered in Guinea and Niger. This interest in integration in Guinea and Niger also reflects the broad mandate and goal of the CMS in each of these countries, as well as external donor interest.

The importance of having participants identify clear and well-understood scenarios while considering the implications on administrative and start-up costs, HR, potential risks, and political will cannot be understated.

### Lesson 3: Modeling provides the evidence but not the answer

Modeling is a tool to weigh the benefits and drawbacks of a complex set of trade-offs and interdependencies among the many components of the supply chain design. It helps evaluate the performance of the various supply chain strategies without having to invest in those changes preemptively [16, 17].

There are limits to modeling. The results of modeling can serve as evidence for answering the “what if” questions; however, the “what’s best” question must still be answered by stakeholders who understand the results of modeling and can place it in the reality of the country context. For example, ignoring administrative boundaries to adjust resupply points for shorter travel distance will reduce operating costs, but the political will to make that change may require significant advocacy and coordination, as noted in Sierra Leone. Additionally, this type of change has implications for health system reporting and supervision structures, administrative policies, and forecasting processes, which cannot be modeled. In short, some changes may be feasible but would require stakeholder input, guidance, and applying common sense to the results of the analysis.

Understanding the possibilities and limits of modeling at the beginning of the system design approach can maximize this approach to identify scenarios that are realistic and produce useful results from the analysis.

### Lesson 4: Costs should not be weighted above other decision criteria

The Traffic Light Analysis tool proved to be an effective way to communicate information on the effects of the different scenarios on all of the identified criteria, while full details of the analysis were provided during the report-back workshop for a more in-depth understanding of the potential changes and requirements. The tool emphasizes the importance of considering both quantitative and qualitative key decision criteria for scenario assessment. While the qualitative criteria are somewhat subjective, they are no less important. For example, the equity criteria depend greatly on the country context yet also reflect the global importance of equity in the supply chain and for immunization coverage [18, 19].

It is also important to link the results analysis to the purposes and priorities of the system design approach as determined by stakeholders during the first step of this approach. For example, some scenarios may have higher operational costs, but support the priority of reducing the vaccine collection burden on health workers. Using this set of criteria helps weigh the trade-offs of design choices and contributes to reaching consensus for prioritizing actions.

### Lesson 5: Data collection—work smarter, not harder

It is not practical or necessary to visit every HF or distribution point in a country to collect primary data from every individual involved in supply chain management. Primary data collected from a sample of facilities and distribution points provide sufficient insight to create generalized cost assumptions to apply across each country model. Stakeholders validated the appropriateness of these cost assumptions during assumption-validation meetings. The key is that because individual costs will vary not only across hundreds or thousands of HFs, but also over time, it is impractical to collect detailed cost data for each point in the supply chain. However, if a small sample can provide cost estimates that are close to correct on average, the resulting model can still help identify the likely results of structural changes to a supply chain. The modeling results therefore provide a high-level overview (such as total supply chain costs) or answers that are “directionally consistent” (i.e., the approach can show which design yields cost increases, but not necessarily the exact expected costs) with insight into some details (such as specific CCE with constraints).

Several challenges emerged during data collection and cleaning. First, inconsistency across different datasets related to facility names and spelling required a significant level of cleaning and matching to create a reliable master list of facilities with their corresponding CCE, locations, and catchment populations. A standardized master facility list for the ministry of health would be beneficial to this approach, but such standardization did not exist in any of the four countries included in this analysis. Secondly, as the system is dynamic, information changes regularly and datasets become outdated quickly. This was particularly true for the CCE inventory as each country was receiving new equipment during the same time period that these analyses were being conducted, and even the number of facilities was changing as facilities opened and closed.

### Lesson 6: Not all questions can be answered with a computer model

Modeling can analyze the complex interplay among many supply chain components and provide insight into how changes can affect supply chain performance. Modeling is best used to determine potential changes in the characteristics and flow of products, and to identify storage locations and needs, transport needs and optimal routes, and inventory needs. Beyond these, modeling can provide indications of HR needs, information management systems and data flow requirements, and equity considerations for vaccine availability, but further and different analyses are required for more precise estimates for these and other aspects of the supply chain.

In the four countries, stakeholders identified a wide range of priorities for the iSC to address with system design and modeling, some of which would need separate analysis and planning. In Madagascar, Guinea, and Niger, stakeholders set the goal of a reliable data-driven supply chain. This is best achieved through a well-functioning logistics management information system and structured data review teams [20]. However, the impact of those components and how well they are managed cannot be captured in a supply chain model. Human resources is another important component of the supply chain, but fully understanding HR needs requires a workforce assessment complemented by a capacity-building strategy to close any gaps. A final example is immunization coverage. Vaccine availability is a requirement for immunization coverage, yet many other factors—accessibility to an HF, caregiver knowledge, financial resources, migration patterns, and even trust in the immunization program and health system—affect a child’s ability to get vaccinated [21]. A computer model can identify the truck and warehouse capacities and optimal delivery frequencies, but only a caregiver can determine if a child arrives at an HF to receive a vaccine.

Additionally, computer modeling cannot determine total actual current supply chain costs. Results of the modeling analysis provide estimated total operating costs and costs per dose delivered within each of the different scenarios; however, these do not reflect financially accurate costs that a costing assessment can provide. In Madagascar, an initial supply chain costing exercise was conducted and the results used to build the supply chain model and contribute to the system design analysis. A costing assessment can identify cost drivers and gaps in funding for specific segments or components of the supply chain, and provide evidence for advocating and planning for funding [22].

## Discussion

A system design approach can identify changes to the design of the supply chain that can introduce efficiencies and improve reliability. The lessons from these four countries contribute to the global thinking and best practices related to system design and its applicability at the country level. As a core principle, stakeholder engagement and consensus building set the foundation for this approach and aligned priorities. This is particularly important as some of these priorities may be inherently conflicting [12]. For example, ensuring direct delivery from the districts to HFs inevitably will introduce incremental costs borne by the district government, compared to a system that uses an ad hoc approach that largely depends on health workers collecting vaccines (and often paying out of pocket for transport). However, it may also contribute to the ultimate goal of improving immunization coverage rates while reducing the burden on the health worker. The system design approach helps weigh the trade-offs with both quantitative and qualitative decision criteria.

Another core principle is selecting scenarios that are considered feasible. Although tools can model many scenarios, only some can be implemented due to resource constraints, policies, and even in-country politics. Scenarios can ask the “what if” questions, but they must reflect the real-world context [23]. For example, integration of vaccines into the CMS is considered a way to improve efficiency [24], but it must be managed appropriately and can often be challenging to get political will for such a significant change in management and product flow. The results from the modeling exercise may provide evidence that will garner the needed political will, or, depending on the country context, be of no help at all.

A limitation of this study is that it does not follow the fifth step of change implementation. While it provides the evidence for change, it remains to be seen how or if stakeholders will advance any changes to the supply chain design. While many of the scenarios present innovative ideas, implementing change will require using the evidence from the system design approach to advocate with key decision-makers.

It is important to note that system design provides illustrative results to guide decision-makers. It does not give a "final answer" but compares and contrasts the trade-offs between the various scenarios. Despite the quantitative evidence gained from modeling, common sense and understanding of the country context must be the basis of system design analysis. Available evidence can inform decision-making to achieve better-performing supply chains.

## Data Availability

The data, data collection tools, analysis and other materials can be obtained from the corresponding author.
